# Upregulation of an IAA-Glucosyltransferase *OsIAGLU* in Rice (*Oryza sativa* L.) Impairs Root Gravitropism by Disrupting Starch Granule Homeostasis

**DOI:** 10.3390/plants14101557

**Published:** 2025-05-21

**Authors:** Guo Chen, Xiaoyu Fu, Xinya Ruan, Xiaolu Yu, Dianyun Hou, Huawei Xu

**Affiliations:** 1 College of Agriculture, Henan University of Science and Technology, Luoyang 471000, China; cguo1010@163.com (G.C.); 13608423875@163.com (X.F.); 19837068271@163.com (X.R.); dianyun518@163.com (D.H.); 2 College of Life Sciences, South China Agricultural University, Guangzhou 510642, China; 13662506485@163.com

**Keywords:** root gravitropism, *OsIAGLU* overexpression, auxin, starch granule accumulation, rice (*Oryza sativa* L.)

## Abstract

Indole-3-acetic acid (IAA) glycosyltransferase (IAGLU) plays vital roles in modulating plant development and responses to environmental cues. Here, we elucidate the regulatory mechanism of *OsIAGLU* in modulating root gravitropism using *OsIAGLU*-overexpressing (OE) rice (*Oryza sativa* L.). *OsIAGLU* upregulation substantially decreases IAA levels, resulting in the impairment of multiple agronomic traits and root gravitropism, as well as nearly complete suppression of starch granule accumulation in rice root tips. Exogenous application of the auxin analog 1-naphthaleneacetic acid (NAA) effectively rescued both starch granule accumulation and root gravitropism. Starch synthesis genes exhibited relatively stable or slightly decreased expression following NAA treatments, whereas all starch degradation genes displayed a consistent downward trend in expression after NAA treatment. This suggests that starch degradation genes may play a more prominent role in regulating starch granule accumulation in rice roots, contrasting sharply with their roles in *Arabidopsis*. Moreover, decreased auxin levels perturbed the accumulation and distribution of hydrogen peroxide (H_2_O_2_) in rice root tips, while NAA treatment restored normal H_2_O_2_ distribution and accumulation in OE roots. This study clearly demonstrates that auxin not only functions in regulating agronomic traits but also plays an essential role in gravity perception by modulating starch granule accumulation in rice root tips.

## 1. Introduction

The phytohormone auxin plays a crucial role in virtually every aspect of plant growth and development, including organ formation, apical dominance, flowering, and numerous other physiological processes [1,2,3,4]. Additionally, auxin mediates responses to various environmental stimuli such as light, low temperature, and gravity [5,6,7,8,9,10,11]. Consequently, the levels and spatial distribution of the active auxin indole-3-acetic acid (IAA), the predominant form of auxin in plants, must be precisely regulated through biosynthesis, transport, conjugation, and catabolism [12,13,14].

Plants have evolved a sophisticated regulatory network to ensure optimal growth by precisely modulating auxin concentrations [15]. The auxin pool comprises several forms, including active auxins, inactive auxins, and auxin precursors [12], with only up to 25% of total IAA being active [16]. Auxin UDP-glycosyltransferase (IAGLU), an enzyme, catalyzes the reaction of free IAA with glucose to generate IAA-glucose, and plays a critical role in modulating active IAA levels in plants [16,17]. The *IAGLU* gene was first cloned from maize (*Zea mays* L.) [18], and, subsequently, an *iaglu* homolog (UGT84B1) was identified in *Arabidopsis* [19]. Overexpression of *UGT84B1* disrupts auxin homeostasis, leading to auxin deficiency phenotypes in transgenic lines [20]. Similarly, another UDP-glucosyltransferase UGT74E2, which catalyzes the transfer of sugars to the auxin indole-3-butyric acid (IBA), is involved in regulating plant architecture and responses to water stress [21]. These findings indicate that *IAGLU* plays a vital role in maintaining auxin homeostasis in plants. In rice (*Oryza sativa* L.), the *OsIAGLU* gene (Os03g0693600), which exhibits high homology with *ZmIAGLU*, was constitutively overexpressed to reduce IAA levels. This resulted in a significant increase in tiller number and panicle formation due to upregulation of *OsIAGLU* [22]. Further analysis revealed that *OsIAGLU* overexpression also leads to an increased leaf angle and impaired root gravitropism [23]. In vitro enzymatic assays demonstrated that OsIAGLU (OsIAGT1) catalyzes the glucosylation of several forms of auxins, including IAA, IBA, indole-3-pyruvic acid (IPA), 1-naphthaleneacetic acid (NAA), 2,4-dichlorophenoxyacetic acid (2,4-D), and indole-3-carboxylic acid (ICA). Overexpression of *OsIAGLU* significantly reduces IAA content and retards plant growth [24]. Additionally, another *OsIAGLU* gene (Os11g0446700) has been demonstrated to regulate root growth [25], seed vigor [26], and submergence tolerance [27]. This underscores the diverse and essential functions of *IAGLU* in plant growth and development.

Gravity serves as a critical environmental cue for land plants, profoundly influencing their growth and development. Plant roots have evolved a directional growth response to the gravity vector, known as root gravitropism, which is essential for securing water and nutrients [28]. Root gravitropism encompasses three distinct phases: gravity perception, signal transduction from the site of perception to the response zone, and curvature of the root in the elongation zone [29,30,31]. Auxin is widely recognized as a key regulator of root gravitropic responses [30,32], with its distribution primarily controlled by auxin efflux carriers PIN-FORMED (PIN) proteins and influx carriers AUXIN RESISTANT1 (AUX1)/LAX family members [33,34]. Gravity sensing primarily occurs in root tips [35,36], where amyloplast sedimentation plays a crucial role in gravity perception. In *Arabidopsis*, auxin modulates the expression of three key starch granule synthesis genes: *starch synthase 4* (*SS4*), *plastidial phosphoglucomutase* (*PGM*), and *adenosine diphosphate glucose pyrophosphorylase 1* (*ADG1*), thereby influencing starch granule accumulation and contributing to gravitational perception [8]. The subsequent sedimentation of amyloplasts triggers gravity signal transduction, leading to gravitropic response in the elongation zone and ultimately causing gravitropic bending [37,38,39]. Extensive evidence indicates that auxin is the primary signaling molecule mediating the gravitropic response. Upon gravistimulation, auxin efflux carriers AtPIN3 and AtPIN7 relocalize to the lower side of columella cells, resulting in auxin redistribution in *Arabidopsis* [40,41]. Additionally, AtPIN2 also plays a significant role in auxin redistribution following gravistimulation [42,43]. Mutation of *OsPIN1b* results in a curly root phenotype in rice, suggesting that *OsPIN1b* is essential for regulating root gravitropism [44]. Similarly, loss-of-function mutations in *OsPIN2* lead to impaired gravitropic responses in root tips, likely due to disrupted auxin transport and distribution in these regions [9]. Collectively, these findings indicate that auxin has dual functions in gravity perception and gravitropic curvature [8]. Additionally, accumulating evidence suggests that various signaling molecules, such as nitric oxide (NO) [45], Ca^2+^ [46,47], reactive oxygen species (ROS) [47,48,49,50], and molecular hydrogen [51], are implicated in root gravitropism, highlighting the complexity of this regulatory mechanism.

Despite extensive investigation into the role of *OsIAGLU* in regulating plant growth and development, the underlying regulatory mechanisms by which *OsIAGLU* modulates root gravitropism remain poorly understood. In this study, we demonstrate that auxin levels are significantly reduced in *OsIAGLU*-overexpressing (OE) rice. This reduction not only impairs key agronomic traits but also markedly decreases starch granule accumulation in rice root tips and severely disrupts root gravitropism. These effects are likely mediated by a starch homeostasis regulatory mechanism distinct from that observed in *Arabidopsis*. Our findings highlight the dual role of auxin in modulating gravity sensing and gravitropic curvature, underscoring its importance in regulating crop agronomic traits.

## 2. Results

### 2.1. Tissue-Specific Analysis of OsIAGLU in Wild-Type (WT) and OsIAGLU-Overexpressing Seedlings

**Figure 1:**
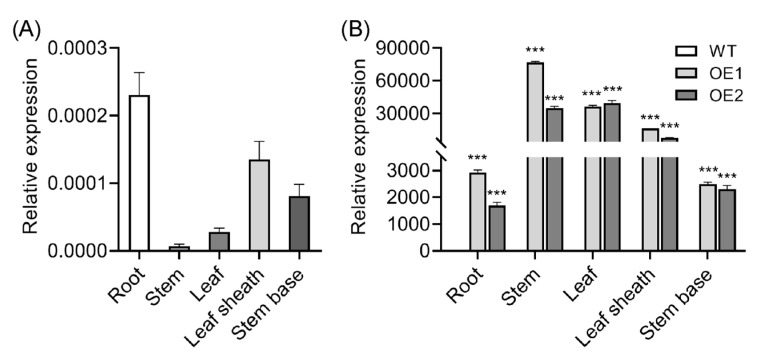
Tissue-specific analysis of *OsIAGLU* in WT and *OsIAGLU*-overexpressing lines. (**A**) Tissue-specific analysis of *OsIAGLU*, data were normalized to the expression of *ACTIN1*. (**B**) Expression analysis of *OsIAGLU* in different tissues of OE lines. The expression level of *OsIAGLU* in WT was defined as 1. Values are means ± standard deviation (SD; *n* = 3). Data were analyzed by ANOVA and Tukey’s tests at *p* < 0.05 significance level. ***: *p* < 0.001.

The expression patterns of *OsIAGLU* in different tissues were analyzed by quantitative RT-PCR (qRT-PCR). The highest transcription levels of *OsIAGLU* were detected in roots, followed by leaf sheaths, stem bases, and leaves, with relatively low levels observed in stems (Figure 1A). This suggests that *OsIAGLU* likely plays a primary role in roots, leaf sheaths, and stem bases rather than in leaves and stems. We subsequently analyzed *OsIAGLU* expression levels in various tissues of overexpression lines. Consistent with the tissue-specific pattern of *OsIAGLU*, the transgenic rice tissues exhibited higher expression levels compared to those in the WT tissues. Notably, the most abundant transcription levels were detected in stems, which showed relatively low expression levels in WT plants. Higher expression levels were also observed in leaves and leaf sheaths, while relatively lower expression levels were found in stem bases and roots (Figure 1B). These results further confirm that *OsIAGLU* is predominantly expressed in rice roots and demonstrate substantial overexpression in all tested tissues of the OE lines.

### 2.2. Overexpression of OsIAGLU Disrupts Auxin Homeostasis

**Figure 2:**
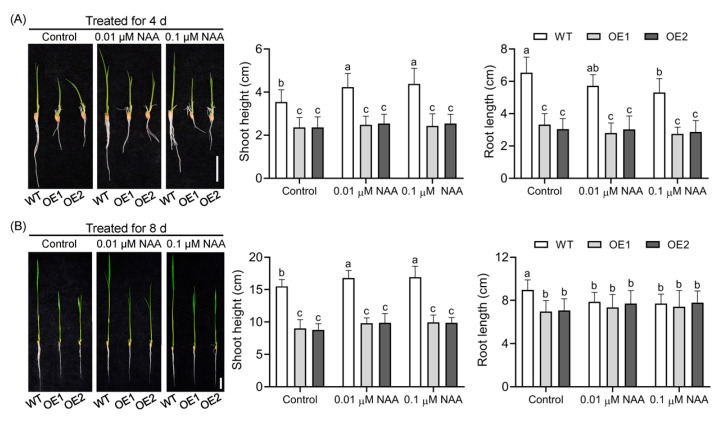
Upregulation of *OsIAGLU* substantially disturbs auxin homeostasis in rice. Phenotype, shoot height, and root length analysis after NAA treated for 4 days (**A**) and 8 days (**B**). Bar = 2 cm. Values are means ± standard deviation (SD; *n* = 16). Data were analyzed by ANOVA and Tukey’s multiple comparisons at *p* < 0.05 significance level. The presence of the same lowercase letter indicates no significant difference between the means (*p* > 0.05).

Overexpression of *OsIAGLU* reduces auxin content in rice lamina joints and root tips [23]. However, the mechanisms by which *OsIAGLU* influences auxin homeostasis across the entire plant remain unclear. To address this, exogenous application of NAA was utilized to investigate auxin homeostasis in transgenic lines. Compared with untreated controls, NAA treatment significantly promoted shoot growth while inhibiting root growth in WT plants (Figure 2). Under normal conditions, upregulation of *OsIAGLU* markedly retarded both shoot and root growth, highlighting its crucial role in regulating rice growth. We observed that the shoot height of WT plants increased more rapidly compared to OE lines under NAA treatment conditions. NAA treatments significantly increased the shoot height of WT plants, while the shoot height of OE lines remained statistically unchanged, suggesting a lower auxin content in the transgenic lines. Compared with the inhibition of root length of WT plants under NAA treatments, the root length of OE lines remained relatively stable upon NAA treatments. The root length of transgenic lines was significantly shorter than that of WT after 4 days of treatment (Figure 2A). However, after 8 days of treatment, root length of OE lines was statistically comparable to that observed in WT (Figure 2B). These results strongly suggest that overexpression of *OsIAGLU* leads to insensitivity to NAA treatment, indicating a potential decrease in auxin levels in the entire transgenic rice plants.

**Figure 3:**
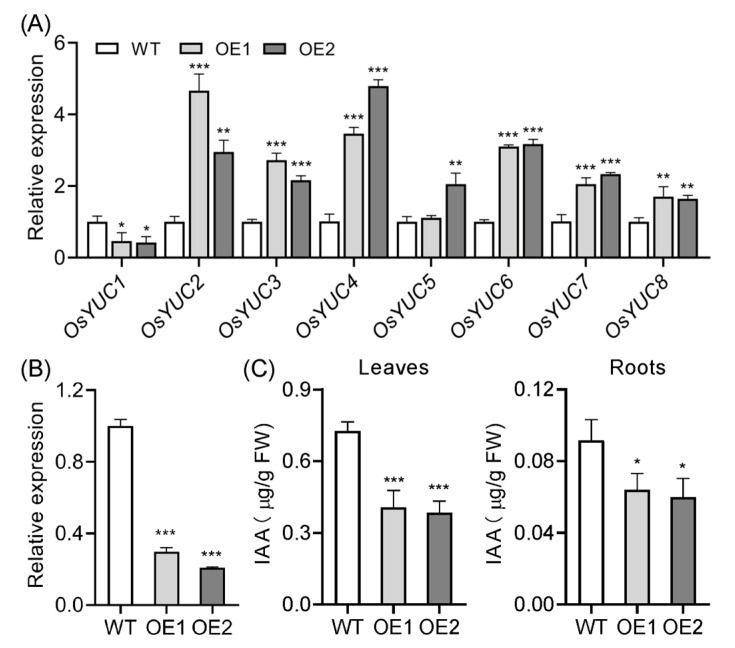
Expression analysis of *OsYUCs* and *OsIAA20*, and measurement of auxin content. (**A**) *OsYUCs* expression analysis in WT and OE lines. (**B**) Relative expression of *OsIAA20* in WT and OE lines. (**C**) IAA content measurement in WT and OE lines. Values are means ± standard deviation (SD; *n* = 3). Data were analyzed by ANOVA and Tukey’s tests at *p* < 0.05 significance level. *: *p* < 0.05; **: *p* < 0.01; ***: *p* < 0.001.

In plants, auxin is primarily synthesized via the tryptophan (Trp)-dependent pathway [2], and the YUCCA (YUC) flavin monooxygenase plays a pivotal role in auxin biosynthesis [52,53]. To investigate the effect of *OsIAGLU* overexpression on auxin biosynthesis, qRT-PCR was employed to analyze the expression levels of *OsYUC* genes in rice roots. Notably, the majority of *OsYUC* genes, which have previously been established as being negatively regulated by auxin [24,54], exhibited upregulation in the overexpression lines, with the exception of *OsYUC1*, which showed a significant decrease in expression (Figure 3A). In addition, a substantial body of evidence demonstrates that *OsIAA20* can serve as a marker gene for evaluating IAA content in plants [10,55,56]. Consistent with this, the expression of *OsIAA20* was significantly reduced in the OE lines (Figure 3B). Furthermore, we utilized a recently developed rapid and robust method for measuring auxin content in transgenic plants [57]. Our results indicated a significant decrease in auxin levels in both transgenic plant leaves and roots (Figure 3C). Collectively, these findings strongly suggest that overexpression of *OsIAGLU* enhances *OsYUC* gene expression and markedly reduces auxin content in rice, indicating a potential metabolic compensation mechanism for auxin deficiency.

### 2.3. Overexpression of OsIAGLU Adversely Impacts Rice Agrinomic Traits

**Figure 4:**
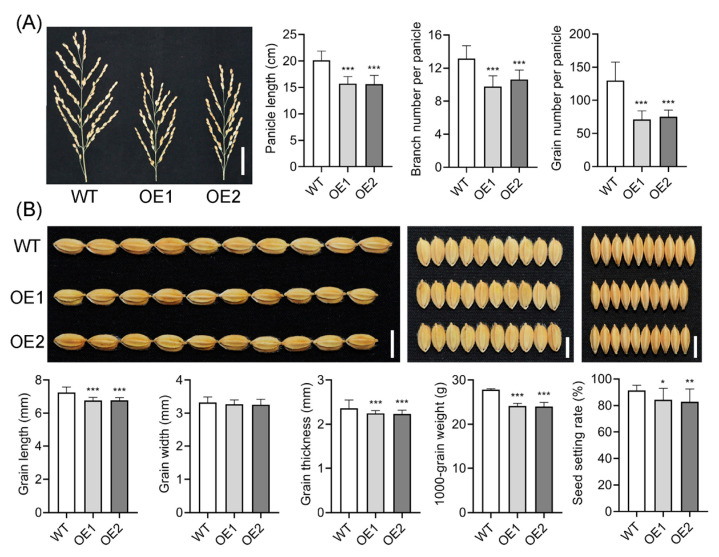
Effect of upregulation of *OsIAGLU* on rice agronomic traits. (**A**) Panicle length, branch number per panicle, and grain number per panicle, bar = 4 cm. (**B**) Grain size, 1000-grain weight, and seed setting rate. Bar = 0.5 cm. Values are means ± standard deviation (SD; *n* = 18). Data were analyzed by ANOVA and Tukey’s tests at *p* < 0.05 significance level. *: *p* < 0.05; **: *p* < 0.01; ***: *p* < 0.001.

Overexpression of *OsIAGLU* results in retarded plant growth, increased tiller number, and wider leaf angles [22,23]. However, how the upregulated *OsIAGLU* influences rice agronomic traits remains poorly characterized. To this end, we conducted a detailed comparison of the agronomic traits between WT and OE lines. Our results showed that both panicle length and the number of branches per panicle were significantly reduced in transgenic lines (Figure 4A). Consistently, the grain number decreased by 42% and 45% in transgenic lines, and the grain weight per panicle decreased by 54% and 55%, respectively. Upregulation of *OsIAGLU* also influenced seed size. Specifically, grain length and thickness were markedly reduced in transgenic lines, whereas grain width remained similar to that of the WT plants (Figure 4B). Consistent with the reduced seed size, the 1000-grain weight was also substantially decreased in OE lines. Furthermore, the seed setting rate was notably impaired in OE lines (Figure 4B). These findings provide strong evidence for the critical role of *OsIAGLU* in regulating rice agronomic traits.

### 2.4. Upregulation of OsIAGLU Impairs Root Gravitropism by Disruping Starch Granule Accumulation in the Root Caps

**Figure 5:**
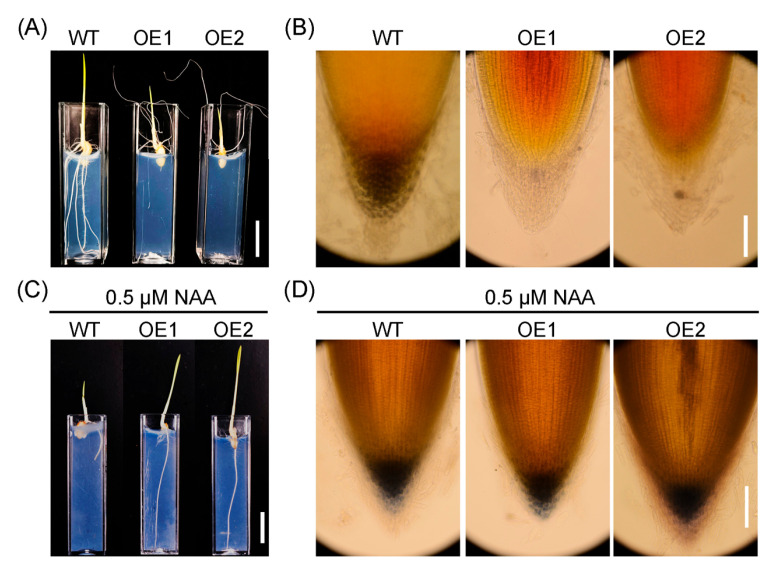
Auxin affects root gravitropism and starch granule accumulation in rice root tips. (**A**) Overexpression of *OsIAGLU* impairs root gravitropism. (**B**) Overexpression of *OsIAGLU* disrupts starch granule accumulation. (**C**) NAA treatment rescues OE root gravitropism. (**D**) NAA treatment restores starch granule biosynthesis in OE lines. (**A**,**C**): Bar = 1 cm; (**B**,**D**): Bar = 0.1 mm.

Transgenic roots cultured on half-strength Murashige and Skoog (MS) agar solid medium exhibited a tendency to grow upward rather than downward into the medium, providing clear evidence that overexpression of *OsIAGLU* significantly disrupts rice root gravitropism (Figure 5A). Auxin content exhibits a positive correlation with starch granule synthesis in *Arabidopsis* root tips [8]. Given the reduced auxin levels analyzed in OE roots (Figure 3C), we further investigated starch granule accumulation in rice root tips. Overexpression of *OsIAGLU* nearly eliminated starch granule accumulation in the columella cells of the rice root cap (Figure 5B), suggesting that *OsIAGLU* plays a crucial role in starch granule formation, likely through its influence on auxin homeostasis. Previous studies have shown that auxin plays a crucial role in modulating root gravitropism and starch granule accumulation in the root apex of *Arabidopsis* [8]. Based on these findings, we hypothesize that the reduction in starch granules in the OE root tip may be attributed to decreased auxin levels. To test this hypothesis, we evaluated the effects of exogenous auxin application on root gravitropism and starch granule accumulation in rice root apices. The application of NAA fully restored root gravitropism in transgenic lines (Figure 5C), thereby confirming that auxin levels are indeed critical for regulating root gravitropism. In addition, NAA treatment obviously inhibited root growth in WT plants but markedly stimulated root growth in transgenic lines (Figure 5C), corroborating the reduced auxin content in OE lines relative to WT plants (Figure 3C). Furthermore, consistent with previous report [8], starch granule accumulation in the root apex of transgenic rice upon NAA treatment was substantially increased and comparable to that observed in WT plants (Figure 5D). These results clearly demonstrated that *OsIAGLU* regulates root gravitropism by modulating starch granule accumulation which is mediated by auxin levels in OE plants.

### 2.5. Starch Biosynthesis Genes Are Upregulated in the OE Root Tips

**Figure 6:**
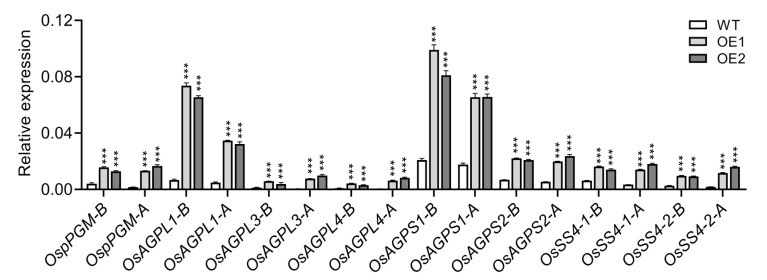
Starch biosynthesis genes expression analysis before and after NAA treatments. Data were normalized to the amplification of the *OsACTIN1* gene. The suffix “-B” following the gene name indicates expression levels before NAA treatment, while the suffix “-A” indicates expression levels after NAA treatment. Values are means ± standard deviation (SD; *n* = 3). Data were analyzed by ANOVA and Tukey’s tests at *p* < 0.05 significance level. ***: *p* < 0.001.

In *Arabidopsis*, auxin positively regulates starch granule synthesis by modulating the expression of several starch synthesis genes rather than starch degradation-associated genes [8]. To investigate whether a similar mechanism exists in rice, we conducted qRT-PCR analysis of starch synthesis genes in rice root tips under both normal and NAA treated conditions. Contrary to the downregulation of starch synthesis genes observed in *Arabidopsis* under reduced auxin levels [8], our results show that all examined starch synthesis genes were significantly upregulated in OE lines, regardless of NAA treatment, compared to those in WT plants (Figure 6). This suggests that rice roots probably possess a unique regulatory mechanism for starch synthesis, which differs from that in *Arabidopsis*. Furthermore, we observed that *OsAGPL1* and *OsAGPS1*, which are involved in modulating phosphorus homeostasis in rice [58], displayed higher expression levels compared to other starch biosynthesis genes before and after NAA treatment, suggesting that these two genes may play a more important role in promoting starch biosynthesis. These findings indicate that the regulatory mechanism governing starch granule accumulation in rice roots likely differs from that in *Arabidopsis*.

### 2.6. Starch Degradation Genes Likely Play a More Prominent Role in Regulating Starch Granule Accumulation in OE Rice Root Tips

**Figure 7:**
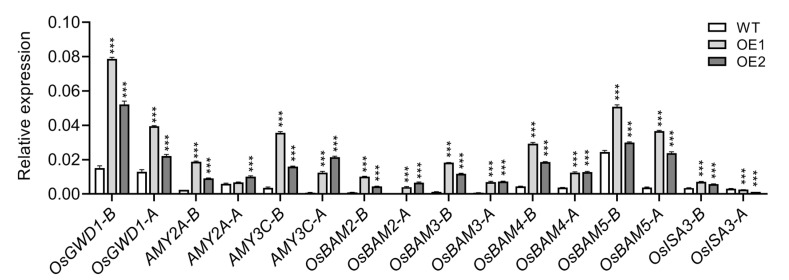
Starch degradation genes detection before and after NAA treatments. Data were normalized to the amplification of the *OsACTIN1* gene. The suffix “-B” following the gene name indicates expression levels before NAA treatment, while the suffix “-A” indicates expression levels after NAA treatment. Values are means ± standard deviation (SD; *n* = 3). Data were analyzed by ANOVA and Tukey’s tests at *p* < 0.05 significance level. ***: *p* < 0.001.

Distinct sets of genes, including those involved in starch synthesis and degradation, work synergistically to regulate starch homeostasis [59,60]. To elucidate the detailed mechanisms underlying starch homeostasis, we performed qRT-PCR analysis to examine the expression levels of genes involved in starch degradation (Figure 7). Similarly to starch biosynthesis genes, most of the tested starch degradation genes exhibited upregulation in response to *OsIAGLU* overexpression, both before and after NAA treatments. Notably, *OsGWD1* and *OsBAM5* showed higher expression levels compared with other genes under identical conditions. Furthermore, all detected genes exhibited consistent downregulation following NAA treatment (Figure 7). Given the significant suppression of starch granule accumulation and the inducible expression of starch synthesis and degradation genes in transgenic root tips, it is plausible that these starch degradation genes play a more critical role in regulating starch granule homeostasis, especially under auxin deficiency conditions. Collectively, exogenous application of NAA effectively rescued starch granule accumulation (Figure 5D), and enhanced the expression of starch biosynthesis and degradation genes (Figure 6 and Figure 7). These findings indicate that starch biosynthesis and degradation genes function coordinately to regulate starch granule production upon NAA treatment, highlighting the complexity of starch homeostasis in rice roots.

### 2.7. Decreased Accumulation of Hydrogen Peroxide (H_2_O_2_) in the Root Elongation Zone Correlates with Reduced Auxin Levels

**Figure 8:**
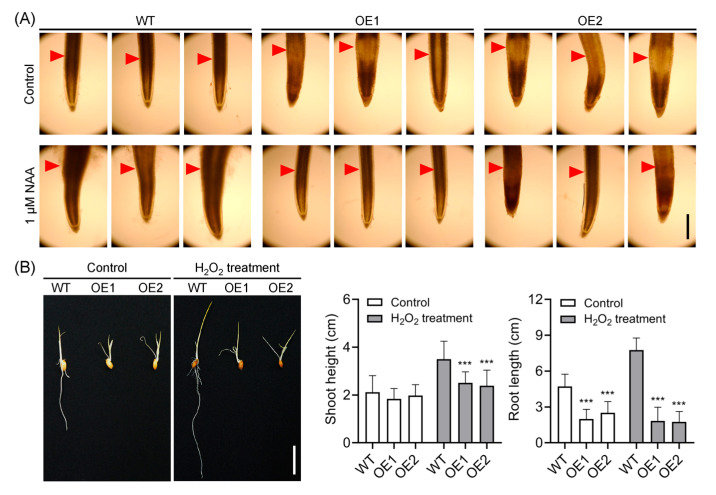
Distribution of H_2_O_2_ upon *OsIAGLU* upregulation and effect of H_2_O_2_ on OE root gravitropism. (**A**) H_2_O_2_ distribution analysis with or without NAA treatments. Red triangle indicates the elongation zone. (**B**) Effects of H_2_O_2_ treatment on seedling growth and root gravitropism. Values are means ± standard deviation (SD; *n* = 32). Data were analyzed by ANOVA and Tukey’s tests at *p* < 0.05 significance level. ***: *p* < 0.001.

It was reported that ROS, particularly H_2_O_2_, play a significant role in regulating root gravitropic bending [49,50,61]. To investigate the involvement of ROS in root gravitropism, we utilized DAB staining to evaluate H_2_O_2_ distribution and accumulation in rice root tips. Consistent with previous findings [49,50], under normal conditions, H_2_O_2_ primarily accumulates in the elongation zone of WT plants, where the bending process initiates [50]. In contrast, H_2_O_2_ accumulation in OE lines was predominantly observed in the meristem zone rather than the elongation zone (Figure 8A), suggesting that impaired root gravity perception also disrupts the distribution of H_2_O_2_ in rice roots. To further investigate the role of H_2_O_2_ distribution in regulating root gravitropism, we evaluated H_2_O_2_ distribution and accumulation following NAA treatment. In line with a previous report [49], NAA treatment obviously enhanced H_2_O_2_ accumulation specifically in the elongation zone of OE roots rather than in the meristem zone. This suggests that auxin is also involved in H_2_O_2_ distribution and accumulation in rice roots. Previous studies have shown that exogenous application of H_2_O_2_ disrupts root gravitropism [62,63], indicating that H_2_O_2_ is implicated in this process. We subsequently examined the role of H_2_O_2_ in regulating root gravitropism. Consistent with earlier reports [64,65], exogenous application of low concentrations of H_2_O_2_ significantly promoted shoot and root growth but did not affect root gravitropism in either WT or OE plants (Figure 8B). These results suggest that the symmetrical distribution of H_2_O_2_, rather than its overall content, is more crucial for regulating root gravitropism.

## 3. Discussion

IAGLU is a crucial enzyme responsible for conjugating free IAA to form inactive IAA. Consequently, IAGLU plays a critical role in regulating auxin homeostasis [23,24]. In this study, several lines of experimental evidence demonstrate that overexpression of *OsIAGLU* in rice leads to a significant reduction in auxin levels. This includes increased resistance to NAA treatment, inducible expression of *OsYUC* genes, reduced *OsIAA20* expression, and indeed lower auxin content in OE lines compared to that in WT plants (Figure 2 and Figure 3). The results demonstrate that overexpression of *OsIAGLU* significantly decreases auxin levels in rice, consistent with previous findings [23,24]. Furthermore, overexpression of *OsIAGLU* significantly impairs multiple agronomic traits, including panicle length, grain size, 1000-grain weight, and setting rate (Figure 4), as well as root gravitropism (Figure 5A). Root gravitropism is closely correlated with IAA content and starch granule accumulation (Figure 5). Both starch synthesis and degradation-related genes play crucial roles in regulating starch granule accumulation in rice root tips (Figure 6 and Figure 7). Despite the reduced auxin levels in OE lines, most *OsYUC* genes exhibited notably increased expression in OE lines compared to WT plants (Figure 3A). Consistent with this observation, previous studies have demonstrated that *YUC* gene expression is regulated via a negative feedback mechanism in response to active auxin levels [66]. Furthermore, an increasing number of studies have suggested that plants have evolved multiple layers of regulatory mechanisms, including transcriptional regulation, post-transcriptional regulation, protein modification, and negative feedback regulation, to maintain auxin homeostasis [67]. Collectively, these findings indicate that precise regulation of auxin levels is essential for ensuring normal plant growth, development, and adaptive responses to diverse environmental stimuli [68].

While auxin has been established as a key regulator in various aspects of plant growth and development, its specific influence on rice agronomic traits remains poorly understood. These transgenic lines serve as suitable models for investigating the role of auxin in regulating plant growth and development, as well as responses to diverse environmental stimuli. We observed that the reduced auxin in OE lines significantly affected several critical agronomic traits, including panicle length, grain size, and seed setting rate (Figure 3), indicating that auxin is essential for rice grain development. Consistent with our findings, deficiency of the rice YUCCA (YUC) flavin-containing monooxygenase encoding gene *OsYUC2* also decreases IAA levels and impairs multiple agronomic traits, such as panicle length, flower number, seed setting rate, and grain weight [69]. Similarly, mutation of *OsYUC11* disrupts auxin biosynthesis and reduces seed size [70]. Further research demonstrated that a novel transcription factor, OsMYB73, modulates starch biosynthesis and grain size by regulating *OsYUC11* expression [71]. Conversely, deficiency of the *dioxygenase for auxin oxidation* (*DAO*) gene results in elevated levels of free IAA and male sterility [72], suggesting that abnormally high auxin levels also impair seed development and lead to parthenocarpy. Collectively, these results highlight the importance of auxin homeostasis during the reproductive stage in regulating seed development. The precise molecular mechanisms underlying auxin homeostasis warrant further investigation.

In addition to its functions in regulating growth and development, auxin is essential for root gravitropism. Auxin is widely recognized as the primary signaling molecule that transmits the gravity signal to the root elongation zone. The asymmetric distribution of auxin between the upper and lower sides of the root leads to differential growth, resulting in root curvature [30]. Although starch-filled amyloplasts function as statoliths in gravity sensing, the precise mechanism governing starch granule accumulation remains unclear. Apart from signal transduction and triggering gravitropic response, Zhang et al. (2019) demonstrated that auxin also regulates starch biosynthesis by fine-tuning three starch granule synthesis genes rather than starch degradation genes in *Arabidopsis* [8]. Additionally, a series of studies reveal that disrupting auxin biosynthesis or signaling decreases starch content in several cereals, such as pea (*Pisum sativum* L.) [73,74], maize [75], rice [70,76,77], and barley (*Hordeum vulgare* L.) [78]. Consistent with our results (Figure 5), a recent report also demonstrated that auxin participates in regulating root gravitropic response by modulating starch granule accumulation in tomato (*Solanum lycopersicum* L.) [79]. Our results further revealed that the expression levels of starch biosynthesis genes in OE lines remain relatively stable upon NAA treatment (Figure 6), whereas starch degradation genes in OE lines are substantially downregulated upon NAA treatment (Figure 7). Therefore, it is reasonable to infer that genes involved in starch degradation may play a more pivotal role in determining starch granule accumulation in rice roots, especially under auxin-deficient conditions. This observation contrasts with the findings reported in *Arabidopsis* [8]. Moreover, although starch granules play a critical role in gravity sensing [31], amyloplasts devoid of starch still elicit a residual gravity response in starchless mutants [31,80,81,82]. This suggests that plastids themselves may be more important for gravity sensing [31,83]. In our study, NAA treatment effectively restored starch granule accumulation and root gravitropism (Figure 5), thereby supporting the starch-statolith hypothesis.

Root gravity perception and gravitropic curvature occur in two distinct zones: the root cap and the elongation zone [30]. In the statocytes of the root cap, amyloplast sedimentation, which is likely the primary mechanism for gravity sensing, initiates a signaling cascade that ultimately leads to gravitropic curvature in the elongation zone [84]. While auxin is recognized as the principal signaling molecule mediating the curvature response to gravitational stimuli, numerous other signaling molecules have also been reported to regulate root gravitropic curvature, such as H_2_O_2_ [30]. Asymmetric distribution of auxin induces root gravitropic curvature in response to gravistimulation, with ROS likely functioning as a downstream component in this signaling pathway [49]. Additionally, exogenous application of H_2_O_2_ has been shown to impair primary root gravitropism and induce root bending in grass pea and pea seedlings during radicle emergence [62,63]. Further investigations showed that H_2_O_2_ treatment increases GA_3_ levels, which subsequently activates α-amylase and leads to starch hydrolysis, thereby disrupting root gravity sensing [48,63]. In the present study, we observed that H_2_O_2_ primarily accumulated in the meristematic zone rather than the elongation zone of OE roots. Similar effects were observed when roots were treated with diphenylene iodonium (DPI), an NADPH oxidase inhibitor, which similarly inhibited root gravitropic curvature [50]. NAA treatment not only restored starch granule accumulation (Figure 4) but also normalized H_2_O_2_ distribution and accumulation in OE roots (Figure 8A), suggesting that H_2_O_2_ may function downstream of auxin and starch-mediated gravity perception. In line with a previous report [64], our results showed that a lower H_2_O_2_ concentration (10 μM) significantly promoted rice growth, and WT plants exhibited faster growth compared to transgenic lines (Figure 8B), implying a relatively lower level of H_2_O_2_ in OE plants. In contrast, unlike previous reports [48,63], we did not observe impaired root gravitropism in WT plants upon H_2_O_2_ treatment, which may be attributed to differences in experimental conditions such as H_2_O_2_ concentration, timing of treatment, and plant species used in our study. Although NAA effectively restored OE root gravitropism and H_2_O_2_ distribution similar to WT plants (Figure 4 and Figure 8), H_2_O_2_ application alone could not rescue OE root gravitropism (Figure 8B). This suggests that H_2_O_2_ acts downstream of auxin to trigger root gravitropic curvature, and that the asymmetric distribution of H_2_O_2_ in root tips, rather than its overall content, plays a crucial role in regulating root gravitropic curvature.

In general, the upregulation of *OsIAGLU* leads to a decline in auxin levels, resulting in impaired agronomic traits and root gravitropism. Consistent with the starch-statolith hypothesis, OE roots exhibit almost complete loss of starch granules in the root caps. Application of NAA effectively restores starch granule biosynthesis and root gravitropism, strongly suggesting that auxin plays a crucial role in regulating root starch biosynthesis and gravitropism. Conversely, despite decreased auxin content and reduced starch granule accumulation, starch biosynthesis genes were significantly upregulated in OE rice root tips, a response distinct from that observed in *Arabidopsis*, indicating a distinct regulatory mechanism between these two species. Furthermore, starch biosynthesis and degradation genes collaboratively regulate starch granule accumulation in response to NAA treatment, underscoring the complexity of the molecular mechanisms governing starch homeostasis. The precise mechanism by which auxin regulates starch granule accumulation in rice root tips requires further investigation. Moreover, ROS act downstream of auxin, and their asymmetric distribution likely plays a crucial role in initiating root curvature. A deeper understanding of the molecular mechanisms underlying ROS signaling will aid in elucidating the nature of root gravitropism.

## 4. Materials and Methods

### 4.1. Plant Lines and Growth Conditions

The OE lines, which were generated based on Dongjin background, were kindly provided by EE Liu (South China Agricultural University). Seed germination and seedling culture were performed according to our previous reports [11,44]. Briefly, WT and transgenic seeds were sterilized with 3% NaOCl for 30 min, followed by washing 3–5 times with distilled water. These seeds were then transferred to Petri dishes and cultured in the dark at 30 °C for 2–3 days. Germinated seeds were subsequently transferred to Kimura B complete nutrient solution [85] for further growth under the following conditions: 30/25 °C (day/night), relative humidity of 60–80%, light intensity of 300 μmol/m^2^/s, and photoperiod of 12 h light/12 h dark.

### 4.2. NAA and H_2_O_2_ Treatment

As one of the synthetic auxins, NAA is more stable than the natural auxin IAA and has been widely used as a substitute for IAA in exogenous application experiments [66]. After 3 days of germination, plant materials were transferred to Kimura B complete nutrient solution containing 0.01 μM or 0.1 μM NAA. Shoot height and root length were measured after 4 and 8 days of treatment to evaluate the effect of NAA on rice growth. For H_2_O_2_ treatment, plant materials were transferred to Kimura B complete nutrient solution, supplemented with 10 μM H_2_O_2_, and treated for 4 days, after which shoot height and root length were measured.

### 4.3. IAA Measurement

IAA content was assayed according to a recent report [57]. Briefly, approximately 0.05 g of 7-day-old seedling leaves or roots were collected and completely homogenized using a multi-sample tissue grinder Tiss-Basic48 (Shanghai Jingxin Industrial Development Co., Ltd., Shanghai, China) helped by liquid nitrogen. In total, 0.3 mL 100% ethanol was added to the powdered sample, which was then centrifuged at 12,000 rpm for 10 min at 4 °C. Next, 100 μL of the supernatant was transferred to 900 μL of assay reagent (water:concentrated sulfuric acid:0.5M FeCl_3_ = 25:15:0.75) and incubated for 30 min at room temperature. The absorbance of the reaction mixture was measured at 540 nm and used for auxin content calculation.

### 4.4. Root Gravitropism Observation

For the gravitropic observation, seeds were placed on the surface of half-strength Murashige and Skoog (MS)-agar solid medium, with or without NAA.

### 4.5. Starch Granule Staining Assay

WT and transgenic rice roots, treated or untreated with NAA, were collected for starch staining. Roots were immerged in an I_2_-KI solution (0.33% (*w*/*v*) I_2_ and 0.67% (*w*/*v*) KI) for 5 min, then rinsed in chloral hydrate (40 g chloral hydrate was dissolved in 10 mL glycerine and 20 mL ddH_2_O) for 5 min. Subsequently, the root tips were observed under a microscope [44].

### 4.6. Quantitative Real-Time PCR (qRT-PCR) Analysis

Quantitative real time reverse transcriptase PCR (qRT-PCR) analysis was performed using gene-specific primers (Table S1) according to the method described previously [11,86]. Root tips about 0.5 cm in length were sampled both before and after 12 h of NAA treatments for the analysis of gene expression related to starch biosynthesis and degradation. The data were normalized to the amplification of the *OsACTIN1* gene (Os03g0718100). For expression analysis of *OsIAGLU* in WT and OE lines, the expression level of *OsIAGLU* in WT was defined as 1.

### 4.7. Expression Analysis of Starch Biosynthesis and Degradation Genes

To investigate the function of starch biosynthesis and degradation genes in regulating starch homeostasis in rice root tips, we retrieved genes involved in these processes from the China Rice Data Center (https://www.ricedata.cn/, accessed on 1 May 2023). These genes involved in starch biosynthesis include *plastidic phosphoglucomutase* (*OspPGM*, Os10g0189100), *ADP-glucose pyrophosphorylase Large Subunits* (*OsAGPL1*, Os05g0580000; *OsAGPL3*, Os03g0735000; *OsAGPL4*, Os07g0243200), *ADP-glucose pyrophosphorylase Small Subunits* (*OsAGPS1*, Os09g0298200; *OsAGPS2*, Os08g0345800), and *starch synthases* (*OsSS4-1*, Os01g0720600; *OsSS4-2*, Os05g0533600). Additionally, six genes involved in starch degradation were obtained: *α-glucan water dikinase 1* (*OsGWD1*, Os06g0498400), α-*amylases isozymes* (*AMY2A*, Os06g0713800; *AMY3C*, Os09g0457800), *β-amylases* (*OsBAM2*, Os10g0465700; *OsBAM3*, Os03g0141200; *OsBAM4*, Os01g0236800; *OsBAM5*, Os10g0565200), and *isoamylase 3* (*OsISA3*, Os09g0469400). The expression changes in these genes in wild-type (WT) and transgenic lines before and after NAA treatments were detected using qRT-PCR.

### 4.8. H_2_O_2_ Staining

3′3-diaminobenzidine (DAB) staining was performed as previously described [87]. After staining, the root tips were observed under the microscope.

### 4.9. Statistical Analysis

All experiments were conducted at least three times with consistent results. Data were expressed as means ± SD and were analyzed by one-way ANOVA in GraphPad PRISM (version 8.0.2) at the significance levels of *p* < 0.05 (*), *p* < 0.01 (**), and *p* < 0.001 (***). Multiple comparisons were performed according to Tukey’s method at *p* < 0.05 significance level. Figures were created by GraphPad Prism 8.0.2.

## 5. Conclusions

Taken together, in this investigation, we elucidated the role of *OsIAGLU* in regulating rice architecture and agronomic traits, with a particular focus on its function in root gravitropism regulation. Our results reveal that overexpression of *OsIAGLU* disrupts auxin homeostasis, thereby negatively impacting rice growth and development. More intriguingly, upregulation of *OsIAGLU* impairs rice root gravitropism. Notably, for the first time, we provide evidence suggesting that starch degradation genes may play a more pivotal role in regulating rice root starch granule accumulation, which is distinct from those reported in *Arabidopsis*. The underlying molecular mechanism by which auxin influences starch granule accumulation remains to be elucidated. Further research into this topic could shed light on the molecular processes underlying rice root gravitropism and identify novel genes for optimizing root architecture, and provide potential strategies for modulating starch accumulation, thus contributing to addressing future food security challenges.

## Data Availability

The original contributions presented in this study are included in the article/Supplementary Materials. Further inquiries can be directed at the corresponding author.

## Supplementary Materials

The following supporting information can be downloaded at: https://www.mdpi.com/article/10.3390/plants14101557/s1, Table S1: Primers used in this study.
