# Supplementary material for: Upregulation of an IAA-Glucosyltransferase OsIAGLU in Rice (Oryza sativa L.) Impairs Root Gravitropism by Disrupting Starch Granule Homeostasis
Source: Plants (Basel). 2025 May 21;14(10):1557. doi: 10.3390/plants14101557 (PMC12114892; doi:10.3390/plants14101557)
Supplement: Supplementary file 1 [file plants-14-01557-s001.zip › plants-3622019-supplementary.pdf]

Table S1 Primers used in this study.

| Primers for qRT-PCR | Primer sequences (5′-3′) |
|---|---|
| OsIAAGLU-qF | TGGACGAGTTCGTGGAGTTC |
| OsIAAGLU-qR | TCACATCTCCGACGCTGCAG |
| OsYUC1-qF | AGGTGTTGGTCGTGGGATGCG |
| OsYUC1-qR | GCGATGCCGAACGTGGATAGA |
| OsYUC2-qF | TATGGATCGGCAACCATTTGA |
| OsYUC2-qR | CGCTGGGAAGACTGTCCTTGT |
| OsYUC3-qF | GGAAGCGTGTTCTCGTTGTTG |
| OsYUC3-qR | ACATTGACAGCCCAAAGGTGG |
| OsYUC4-qF | CCTCGACCTCTGCAACCACAATG |
| OsYUC4-qR | CGACAACAGGAGTACCAGCCAATC |
| OsYUC5-qF | GTCAGCCTCGACCTCTGCAACA |
| OsYUC5-qR | TGGGAAACCACTTGAGAAGGAACAC |
| OsYUC6-qF | GGATACCAAAGCAACGTCCCC |
| OsYUC6-qR | TGAAGCCAACAGAGTAGAGCCCTG |
| OsYUC7-qF | ACCGGCTACCGCAGCAATGTG |
| OsYUC7-qR | CGTACAGCCCCGACTCACCCT |
| OsYUC8-qF | GAGATGTGCCTGGACCTCTGC |
| OsYUC8-qR | GTGTCTCCCAGCACCATCCTT |
| OsIAA20-qF | TGGCGGATATGTGAAGGTGAA |
| OsIAA20-qR | TATGAGCCGAGGATGGACAAG |
| OspPGM-qF | GTGGTGATGGCCGATACTTTA |
| OspPGM-qR | GTTCCTGCCAACTAGGATCTTC |
| OsAGPL1-qF | AGCTGCCCCGAGTGAAGTAG |
| OsAGPL1-qR | ACACATGAGATGCACCAACGA |
| OsAGPL3-qF | GGTGGAATAAAGCCCTCCCTTA |
| OsAGPL3-qR | CTGCCGCAAATGGTCATG |
| OsAGPL4-qF | CAGATATGAGTTCGGCGTCTACTC |
| OsAGPL4-qR | GGCGGGTTCAGCTTTGG |
| OsAGPS1-qF | TCCAGTGCACATCAAGCAATC |
| OsAGPS1-qR | GCTTGGTGAACTGGCAGCAT |
| OsAGPS2-qF | GATACCGTGCTTGAATTTTCCAT |
| OsAGPS2-qR | TGGGATTGCTGGATTCATTTTC |
| OsSS4-1-qF | CACTTGCCACAACTTCGAATAC |
| OsSS4-1-qR | CTTGCATCCTGTCTGGTCTATC |
| OsSS4-2-qF | GTGCAGAAGGACATGAGGATAG |
| OsSS4-2-qR | GAGCCACTGCTCTTTGATAGAT |
| OsGWD1-qF | GCGAAATACGGAAAACTGTTCTC |
| OsQWD1-qR | GTTGGTCACCTTCATCTCCAG |
| AMY2A-qF | GCCGATCATCGCACCTCTTC |
| AMY2A-qR | CGATCCCACATATCAGTGACG |

| | |
|---|---|
| AMY3C-qF | AGACTTCCATGTCGTTGCTC |
| AMY3C-qR | CAGGCACAAAATAGTCCTG |
| OsBAM2-qF | CCTTTTGGGGTGGTTCATCTG |
| OsBAM2-qR | GCCATCGTGTAGAAAATCTTAACCCG |
| OsBAM3-qF | CGCCTGTCTGTCTCTGTTTGATTGATTC |
| OsBAM3-qR | TGAGCCAGGTTGAGCGACATC |
| OsBAM4-qF | GAACAGGTTCACCAAGTTCGTCAG |
| OsBAM4-qR | CCGACATCGAGGAGAGGTAATG |
| OsBAM5-qF | TAGTATGGAGGCGAGCTTCGTGGC |
| OsBAM5-qR | CTGCAAACTCCCCGCATAATC |
| OsISA3-qF | ACAGCTTGAGACACTGGGTTGAG |
| OsISA3-qR | GCATCAAGAGGACAACCATCTG |
| OsACTIN1-qF | CTTCATAGGAATGGAAGCTGCG |
| OsACTIN1-qR | CACCTTGATCTTCATGCTGCTA |
